# Euiiyin-tang in the treatment of obesity: study protocol for a randomised controlled trial

**DOI:** 10.1186/s13063-017-2039-8

**Published:** 2017-06-21

**Authors:** Chunhoo Cheon, Soobin Jang, Jeong-Su Park, Youme Ko, Doh Sun Kim, Byung Hoon Lee, Hyun Jong Song, Yun-Kyung Song, Bo-Hyoung Jang, Yong-Cheol Shin, Seong-Gyu Ko

**Affiliations:** 1 Department of Korean Preventive Medicine, Graduate School, Kyung Hee University, Seoul, Republic of Korea; 2 KM Fundamental Research Division, Korea Institute of Oriental Medicine, Daejeon, Republic of Korea; 3 Department of Korean Preventive Medicine, Semyung University, Chungbuk, Republic of Korea; 4 Department of Applied Korean Medicine, Graduate School, Kyung Hee University, Seoul, Republic of Korea; 5 Department of Korean Medicine Rehabilitation, College of Korean Medicine, Gachon University, Incheon, Republic of Korea

**Keywords:** Euiiyin-tang, Obesity, Sasang constitution, Herbal medicines, Randomised controlled trial

## Abstract

**Background:**

Obesity is a public health concern in many countries due to its increasing prevalence. Euiiyin-tang is an herbal medicine formula often used as a clinical treatment for obesity. It acts to eliminate humidity and purify the blood, the causes of obesity identified by the theoretical framework of Korean medicine. The purpose of this study is to evaluate the efficacy and safety of Euiiyin-tang in treating obesity.

**Methods/design:**

This study is a randomised, double-blinded and placebo-controlled, multicentre trial. It has two parallel arms: the Euiiyin-tang group and the placebo group. A total of 160 obese adult women will be enrolled in the trial. The participants will be randomly divided at a 1:1 ratio at visit 2 (baseline). The participants will be administered Euiiyin-tang or placebo for 12 weeks. The primary endpoint is the change in weight occurring between baseline and post-treatment. The secondary outcomes include average weight reduction, changes in body fat, waist and hip circumferences, body mass index, and lipid profile, and the results of questionnaires such as the Korean version of Obesity-related Quality of Life, the Korean version of Eating Attitudes Test, the Social Readjustment Rating Scale, and the Stress Reaction Inventory.

**Discussion:**

The present study will provide research methodologies for evaluating the efficacy and safety of Euiiyin-tang in patients with obesity. In addition, it will provide evidence of correlation between obesity and Sasang constitutional medicine.

**Trial registration:**

ClinicalTrials.gov, NCT01724099. Registered on 2 November 2012.

**Electronic supplementary material:**

The online version of this article (doi:10.1186/s13063-017-2039-8) contains supplementary material, which is available to authorized users.

## Background

Obesity is defined as a modern disease, associated with diseases such as diabetes mellitus, hyperlipidaemia, and hypertension [1]. The prevalence of obesity (body mass index (BMI) ≥25) in Korean adults is 30.6% according to the third National Health and Nutrition Survey report [2]. Recent epidemiologic data show that the prevalence of obesity has increased, rising from 26.7% in 1998 to 30.9% in 2009 [3]. Consequently, the risk of mortality from coronary heart disease and haemorrhagic stroke mortality has increased [4]. The socioeconomic burdens associated with people who are overweight and/or obese are rising, with a cost of 1787 million US dollars in Korea in 2005 [5]. With the need for anti-obesity agents increasing, it is reported that agents such as sibutramine and rimonabant can cause adverse effects [6]. However, herbal medicine has attracted public attention due to the perception that it has fewer adverse effects.

Euiiyin-tang is a Korean medicine formula. It consists of seven herbs: *Ephedra sinica Stapf* (1.33 g), *Angelica gigantis Radix* (1.33 g), *Atractylodis rhizoma alba* (1.33 g), *Coicis Semen* (3.33 g), *Cinnamomi cortex* (1.00 g), *Paeoniae radix alba* (1.00 g), and *Glycyrrhiza uralensis* (0.67 g).

The primary constituent herbs of Euiiyin-tang have effects on obesity-related factors. Euiiyin (*Coicis Semen*) inhibits reactions of dihydroxyphenylacetic acid (DOPAC), 5-hydroxytryptamine (5-HT), and 5-hydroxyindoleacetic acid (5-HIAA) in the hypothalamus induced by fasting stress [7]. Consequently, Euiiyin helps to reduce hunger caused by dietary restrictions. In addition, Euiiyin has been reported to decrease total cholesterol and triglycerides in blood lipids [8, 9]. Ma-Huang (*Ephedra sinica Stapf*) has been frequently used in the treatment of obesity. Ma-Huang significantly decreases blood glucose, triglyceride, and free fatty acid levels in obese mice, and it has also been shown to reduce BMI, fat percentage, and total cholesterol and triglyceride levels in human studies [10, 11]. Baekchul (*Atractylodis rhizome alba*) inhibits corticosterone increase induced by fasting stress [12].

The effects of Euiiyin-tang include eliminating humidity, purifying blood, and relieving arthralgia according to the theoretical frameworks of Korean medicine [13, 14]. Korean medicine describes the cause of obesity as dampness-phlegm, which refers to the congestion of body fluids [15]. Euiiyin-tang helps to remove dampness-phlegm and circulate body fluids; therefore, it is suitable as a treatment for weight loss.

Sasang constitutional medicine is recognised as a distinct field of Korean medicine separate from that of traditional Chinese medicine. Sasang constitutional medicine divides human beings into four separate categories, or constitutions, based on their physiological characteristics. The prevalence of both general and abdominal obesity is highest among one of these constitutions, the Taeum-in. In addition, members of Taeum-in have a lower basal metabolic rate (BMR) [16, 17]. Sasang constitutional medicine has matched herbal medicines to each constitutional type. Euiiyin-tang contains Euiiyin (*Coicis Semen*) and Ma-Huang (*Ephedra sinica*), two herbs typically matched to Taeum-in [18]. We will examine the Questionnaire for Sasang Constitution Classification II (QSCCII) to identify whether Euiiyin-tang has a significant effect for Taeum-in or not [19, 20]. However, QSCCII was not considered as a primary outcome measure in the study, as it is not yet highly validated.

Euiiyin-tang has been frequently used in obesity treatment in Korean clinics and hospitals. A previous animal study revealed the weight loss effects of Euiiyin-tang; however, upon checking trial registries and a systematic review on the subject, human studies examining the effects of Euiiyin-tang on obesity have not been reported [21]. It has been proved, however, that Euiiyin-tang decreases body weight, blood lipids, and insulin levels in high-fat diet-induced obese mice [22].

The evidence on whether Euiiyin-tang affects weight loss is still insufficient; therefore, it is necessary to conduct clinical trials on this medicine. To address this issue, we designed a randomised, placebo-controlled clinical trial to evaluate the efficacy and safety of the pharmacological intervention, Euiiyin-tang, for weight loss in adult female Korean patients with obesity. We also want to verify the correlation between obesity and Sasang’s Taeum constitution.

## Methods/design

### Study design

**Fig. 1:**
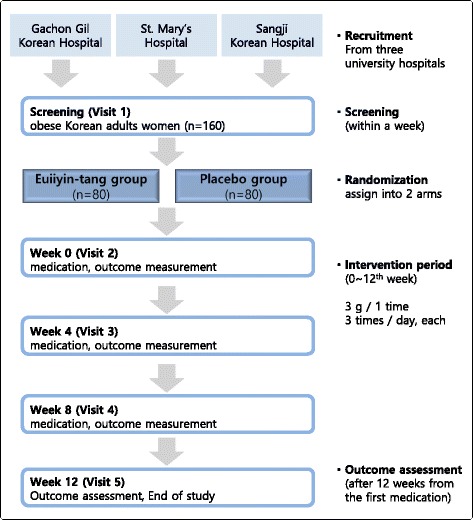
Flow chart of the Euiiyin-tang clinical trial

The present study is a randomised, double-blinded, parallel-group, placebo-controlled multi-centre trial taking place at the following three hospitals: Gachon University Gil Medical Center in Incheon, Seoul St. Mary’s Hospital, and Sangji University Korean Medicine Hospital in Wonju. Participants will be outpatients of these institutions. Any participant fulfilling the eligibility criteria will be included in the study. This study will have two arms: the Euiiyin-tang treatment group and the placebo treatment group. Figure 1 presents an overview of the trial. Protocol amendments are not expected. However, if necessary, any modification to the protocol will be reported to the entire investigational team through a conference. All changes will be included in the final manuscript prior to journal submission.

### Study objectives

The main objectives of the present study are (1) to evaluate the efficacy and safety of Euiiyin-tang in obese Korean female adults and (2) to detect the correlations between obese participants and the Taeum classification of Sasang constitutional medicine.

### Recruitment

Subjects will be recruited through advertisements. Detailed information on the study including study duration, purpose of the study, eligibility criteria, and the intervention is posted on bulletin boards both inside and outside the hospitals, and patients who visit the trial site will do so voluntarily.

### Participants

#### Inclusion criteria

The inclusion criteria of the trial are provided in Table 1.

**Table 1:** Inclusion criteria

Criteria for inclusion of participants
1. Women aged 18–65 years
2. Patients who have any of the following conditions:
2.1. BMI 30 kg/m^2^ or over
2.2. Obese patients, BMI 27–29.9 kg/m^2,^ who have more than one of the risk factors such as hypertension, diabetes, and hyperlipidaemia, and each of the conditions shall be subject to the following standards.
2.3. Hypertension: Patients under adequate treatment after being diagnosed with hyperlipidaemia (BMI 27–29.9 kg/m^2^) and with their blood pressure adjusted (satisfying all the conditions of systolic blood pressure (SBP) ≤145 mmHg and diastolic blood pressure (DBP) ≤95 mmHg)
2.4. Diabetes: Patients with insulin-independent diabetes mellitus adjusting fasting blood glucose <7.8 mmol/L (140 mg/dL) (BMI 27–29.9 kg/m^2^)
2.5. Hyperlipidaemia: Patients under adequate treatment after being diagnosed with hyperlipidaemia (BMI 27–29.9 kg/m^2^) or with measurements of total cholesterol of more than 236 mg/dL or triglycerides of more than 150 mg/dL at screening
3. Subjects who have agreed to a low-calorie diet during the trial
4. Patients who have provided signed written consent for the trialform for

#### Exclusion criteria

The exclusion criteria of the trial are provided in Table 2.

**Table 2:** Exclusion criteria

Criteria for exclusion of participants
1. Patients with endocrine disease such as hypothyroidism, Cushing’s syndrome, etc., conditions that could affect patient weight
2. Patients with heart disease (heart failure, angina pectoris, and myocardial infarction)
3. Patients with uncontrolled hypertension (SBP >145 mmHg or DBP >95 mmHg
4. Patients with malignant tumour or lung disease
5. Patients with cholelithiasis
6. Patients with severe renal disability (serum creatinine (SCr) > 2.0 mg/dL)
7. Patients with severe liver disability (2.5 times higher than the maximum value in normal group on alanine transaminase (ALT), aspartate aminotransferase (AST), alkaline phosphatase (ALP))
8. Patients with insulin-independent diabetes mellitus, with fasting blood sugar more than 7.8 mmol/L (140 mg/dL)
9. Patients with narrow angle glaucoma
10. Patients with history of neurological or psychological disease or currently suffering from such diseases (schizophrenia, epilepsy, alcoholism, drug addiction, anorexia, bulimia, etc.)
11. Patients with history of stroke or temporary ischaemic cardioplegia
12. Patients with history or existence of eating disorder such as anorexia nervosa or bulimia nervosa, etc.
13. Patients with experience of medications that could have an effect on weight within last 3 months such as appetite suppressant, laxative, or oral steroid, thyroid hormone, amphetamine, cyproheptadine, phenothiazine, or medications affecting absorption, metabolism, and excretion
14. Patients under administration of central nervous system or central active weight reduction medications
15. Patients with experience of β-blocker or diuretic as hypertension medication within last 3 months
16. Forbidden treatments (insulin, hypoglycemic agent, antidepressant, antiserotonin agent, barbiturate, antipsychotic, medication abuse concerns)
17. Patients having difficulty with body measurements due to anatomical change such as resection
18. Patients with experience of surgical history for weight reduction; bariatric surgery, etc.
19. Patients judged unable to follow instructions of the trial by clinical trial investigators
20. Women of childbearing age who are pregnant, breastfeeding, planning a pregnancy, or who do not agree to use proper contraceptive methods (birth control pills, hormone implant, intrauterine device (IUD), spermicide, condom, abstinence, etc.). (Women of childbearing age refers to those within 2 years of menopause who have not had a hysterectomy, bilateral tubal ligation, bilateral oophorectomy, etc.)
21. Patients who have used other test products within the past month
22. Patients who have experienced a 10% weight reduction within the past 6 months
23. Those who decided to stop smoking within the last 3 months or who have an irregular smoking habit

### Subject withdrawal criteria

The subject withdrawal criteria are as follows: detection of eligibility violations; occurrence of a serious adverse event; subject has an acute reaction (such as allergy or shock) due to administration of the investigational product; detection of a systemic disease that was not discovered at the screening stage; less than 70% compliance; subject’s withdrawal of consent; use of any forbidden medication or treatment during the trial that could affect the study result; uncooperative subject; occurrence of other significant protocol violations; subject who cannot follow up; subject unable to progress because of worsening of pre-existing disease; investigator’s decision to terminate the process for the sake of the subject’s health. The participants who are withdrawn after randomisation will be followed up to examine outcomes.

### Sample size

The sample size calculation is based on weight change between baseline (visit 2) and after treatment (visit 5). The hypothesis is as follows:

$$ {\mathrm{H}}_0:\updelta =\Delta 1\hbox{-} \Delta 2=0 $$

$$ {\mathrm{H}}_1:\updelta =\Delta 1\hbox{-} \Delta 2\ne 0 $$

where Δ1 = Weight change between visit 5 and baseline in Euiiyin-tang group

Δ2 = Weight change between visit 5 and baseline in placebo control group.

In a study by Heymsfield et al. [23], the standard deviation (SD) in weight reduction of the groups is 3.9. We assume that both groups will have an equivalent SD. The effect size of this study is hypothesised to be 0.5 as the moderate effect. Consequently, the difference in weight change between groups is assumed to be 1.95. The allocation ratio is 1:1. The following formula is used to estimate the sample size [24]:

$$ \mathrm{m}=\left(\frac{1+\psi}{\psi}\right)\kern0.24em \frac{{\left({\mathrm{z}}_{1\hbox{-} \frac{\upalpha}{2}}+{\mathrm{z}}_{1\hbox{-} \beta}\right)}^2}{\varDelta^2}+\frac{{{\mathrm{z}}^2}_{1\hbox{-} \frac{\upalpha}{2}}}{2\left(1+\psi \right)} $$

where *m* = the sample size of one group

$$ \uppsi =\mathrm{the}\;\mathrm{allocation}\;\mathrm{ratio} $$

$$ \upalpha =0.05,\upbeta =0.8 $$

After taking into account the dropout rate of approximately 20%, the sample size for each group is 80 participants, to allow the test to remain within 5% of the two-sided significance level and possess 80% statistical power. Therefore, the total sample size of this trial is 160.

### Randomisation and blinding

The statisticians of Kyung Hee University Centre for Clinical Research and Drug Development (KCRD), the Contract Research Organisation (CRO), will generate random sequences using the SAS program. KCRD will send the random number cards to each institution. Randomisation procedures will be performed at visit 2, using a random number card in a black envelope. The subjects in this trial will be assigned to one of two groups with an allocation ratio of 1:1. All participants, investigators, and monitors will be blinded. No one, with the exception of the statisticians at KCRD, will be aware of which random number refers to either the treatment group or the placebo group. The randomisation table kept in the opaque sealed envelope by the CRO should be opened according to Standard Operating Procedures (SOPs).

### Treatment protocol

The participants will receive either Euiiyin-tang or a placebo drug for 12 weeks. They will be orally administered 3 g of treatment granules with water three times a day after meals for 12 weeks. The daily dosage adheres to guidelines established by the Ministry of Food and Drug Safety (MFDS) regarding dosage and administration of Euiiyin-tang. The participants will be required to return any drug remains in order to calculate compliance. During the trial, the participants will be prohibited from receiving other weight loss treatment.

### Interventions

Euiiyin-tang is a traditional preparation in Korean medicine. It has been approved as a Korean herbal medicine by the Korean MFDS. The intervention consists of 1.7 g Euiiyin-tang soft extract (including 1.33 g *Ephedra sinica stapf*, 1.33 g *Angelica gigantis radix*, 1.33 g *Atractylodis rhizoma alba*, 3.33 g *Coicis Semen*, 1.00 g *Cinnamomi cortex*, 1.00 g *Paeoniae radix alba*, and 0.67 g *Glycyrrhiza uralensis*), 1.15 g lactose hydrates, and 0.83 g corn starch. Euiiyin-tang is categorised as ‘Other analgesics and antipyretics’ by Anatomical Therapeutic Chemical (ATC) code N02BG. The placebo medicine consists of lactose, corn starch, and food colourings, and possesses an appearance, shape, weight, taste, and colour similar to Euiiyin-tang. The treatment drug and placebo will be supplied by Hanpoong Pharm & Foods Co., Ltd.

### Primary outcome measurement

The primary outcome in the present study is the change in weight of participants between baseline (visit 2) and post-treatment (visit 5).

### Secondary outcome measurements

**Fig. 2:**
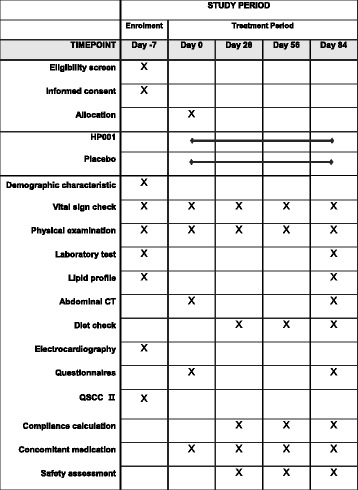
SPIRIT schedule for the trial

The secondary outcomes include the average weight reduction during the visits and among the groups, and changes in body fat, waist and hip circumferences, and waist-to-hip ratio (WHR). In addition, the secondary endpoints include changes in BMI, lipid profile, C-reactive protein (CRP), visceral fat, and glucose, and questionnaire responses to the Korean version of Obesity-related Quality of Life (KOQOL) [25], the Korean version of Eating Attitudes Test KEAT-26 [26], the Social Readjustment Rating Scale (SRRS) [27], and the Stress Reaction Inventory (SRI) [28]. The QSCCII responses will also be assessed. The Standard Protocol Items: Recommendations for Interventional Trials (SPIRIT) study schedule is detailed in Fig. 2.

### Safety outcomes

All variables related to the safety assessment of the study such as vital signs, physical examination, and various laboratory test results, including haematologic test, biochemical test, and urine test, as well as adverse events will be documented on the case report form (CRF) at every visit.

### Statistical analysis

#### Efficacy assessment

The demographic data at the baseline will be analysed using a *t* test for continuous data and a chi-square test for categorical data. Analysis of the primary endpoint will be performed using intention-to-treat (ITT) datasets. Per-protocol (PP) datasets will also be analysed as a reference. The term ITT dataset refers to a set of data regarding all randomly assigned participants. Missing data will be assigned using the last-observation-carried-forward (LOCF) analysis method. PP datasets include data on only the participants who completed the trial with no protocol violations or deviations.

The secondary endpoint data will be analysed using both ITT and PP datasets. The average weight change before and after treatment will be analysed using a paired *t* test for intragroup data and a Student’s *t* test for intergroup data. The average weight during the treatment phase will be analysed using repeated measures analysis of variance (ANOVA) tests. Other secondary data will be analysed identically to the average weight change, with the exception of glucose measurements and questionnaire results, which will be compared only before and after treatment between the groups. The present study has no plan for an interim analysis.

#### Safety assessment

The participants are instructed to report any adverse events which may occur while taking the interventions. All possible adverse events will be described in the CRF. The investigator will withdraw any participant experiencing a serious adverse event. This will be reported to the MFDS within 15 working days of withdrawal according to the Korea Good Clinical Practice (KGCP) guidelines. Participants who have withdrawn will receive appropriate treatment. The investigator will have full responsibility for the safety of participants. For safety assessments, a liver function test, blood cell count test, physical examination, and urine analysis will be conducted at baseline and at visit 5. Any loss caused by the trial will be reimbursed via insurance. The safety datasets will include the participants who are administered Euiiyin-tang or placebo at least once. The incidences of physical examination and self-reported adverse events will be compared using a chi-square test. A liver function test, blood cell count test, and urine analysis will be compared between the groups. The data will be categorised into either normal or abnormal groups according to their respective normal ranges. Differences between the groups will be assessed via a chi-square test.

### Data and safety monitoring

The KCRD will monitor the clinical trial. The monitoring will begin once the first participants complete the required number of visits. All investigational institutions will be monitored based on the SOPs while this trial is in process. Auditing is not scheduled for the present study. To improve data quality, range checks for data values and double data entry will be conducted. Any other committee such as a coordinating centre, steering committee, or endpoint adjudication committee will not be applicable to the present study.

### Ethics and dissemination

This trial is conducted to comply with the Declaration of Helsinki 2008 and/or the regulations of the GCP principles in the Korean MFDS. This trial has received the approval of the institutional review boards (IRBs) of all three institutions: the IRB of Gil Korean Medical Hospital, Gachon University (11–105), the IRB of the Catholic University of Korea Seoul St. Mary's Hospital (KIRB-00393-002), and the IRB of Korean Medical Hospital of Sangji University (SJ IRB 120607). The current protocol is version 1.5, and it is developed according to the SPIRIT checklist (see Additional file 1). All items have been drawn from the World Health Organisation (WHO) Trial Registration Data Set (see Additional file 2). Written informed consent will be obtained prior to the study by the investigator (see Additional file 3). Moreover, we have contracted liability insurance for patient safety. The confidentiality of personal information will be ensured. Each participant will be assigned a trial identification number at enrolment. For the duration of the entire trial, data will be handled based on the trial identification number. All records will remain secure in a locked cabinet or password-protected computer files, both for the duration of the trial and after the trial has concluded. Only investigators will retain the right to access the data. The results of the present study will be disseminated through scientific journals or scientific conference presentations. So far, no public access to the full protocol, participant-level datasets, or statistical code is planned.

## Discussion

The present study investigates the clinical efficacy and safety of Euiiyin-tang in the treatment of patients with obesity. Currently, the increasing prevalence of obesity is a public health concern in many developed countries [29]. The increasing prevalence of obesity is a consequence of social changes leading to the increased consumption of high calorie foods and a reduction in physical activity [30].

Some medical surgeries, such as liposuction and gastric banding, and pharmaceutical agents such as sibutramine, are attributed to certain complications and side effects. Consequently, many patients with obesity seek non-invasive and safer alternative treatment. Therefore, herbal medicine made from natural products has received noticeable attention in obesity treatment [31, 32]. Among these medicines, Euiiyin-tang has been attributed to weight loss in clinical practice [33], but there is little evidence to support this. We obtained an Investigational New Drug (IND) approval from the MFDS to conduct the present clinical trial on Euiiyin-tang.

A previous study on Taeumjowi-tang in patients with obesity resulted in a statistically insignificant primary outcome [31]. In the study, the primary endpoint was the percentage of participants with a weight loss of 5% compared to baseline. However, the study used a strict standard, and consequently did not produce a meaningful conclusion. Therefore, we compared weight changes after the administration of the treatment in each group. The SD value was obtained from Heymsfield et al. [23]. The effect size of 0.5 was derived based on agreement between researchers.

Herbal medicines have been used to treat obesity over long periods of time. However, as the duration of a clinical trial increases, the participant dropout rate increases likewise. As a result, we decided to conduct the follow-up after 12 weeks to confirm significance and to minimise the dropout rate. The study period was adapted from Heymsfield and the previous study on Taeumjowi-tang [23, 34].

Although the present study was limited by permitting only adult women as participants, it nonetheless has several distinctive features. First, it is the only randomised, controlled trial thus far to investigate the efficacy of Euiiyin-tang in the treatment of obesity. In the present study, we will assess not only weight reduction, but also the quality of life and stress through questionnaires. Second, this study will investigate the correlation between obesity and Sasang constitutional medicine, which is a distinct concept of Korean medicine.

In conclusion, the purpose of the present study is to evaluate the efficacy and safety of Euiiyin-tang, which is often used to treat weight loss. We expect that the present study will act as a reference for designing further clinical trials to assess herbal medicine in the treatment of obesity.

## Trial status

Participant recruitment began on 26 April 2013, and 149 participants have been recruited thus far.

## Additional files

Additional file 1: SPIRIT checklist. Standard Protocol Items: Recommendations for Interventional Trials. (PDF 57 kb)

Additional file 2: WHO Data Set. Trial information according to WHO Trial Registration Data Set. (PDF 104 kb)

Additional file 3: Informed Consent Form. Example of Informed Consent Form for Euiiyin-tang trial. (PDF 69 kb)

## Abbreviations

5-HIAA: 5-Hydroxyindoleacetic acid
5-HT: 5-Hydroxytryptamine
ANOVA: Analysis of variance
ATC: Anatomical Therapeutic Chemical
BMI: Body mass index
BMR: Basal metabolic rate
CRF: Case report form
CRO: Contract Research Organisation
CRP: C-Reactive protein
DOPAC: Dihydroxyphenylacetic acid
IND: Investigational New Drug
IRB: Institutional review board
ITT: Intention-to-treat
KCRD: Kyung Hee University Centre for Clinical Research and Drug Development
KEAT-26: Korean version of Eating Attitudes Test
KGCP: Korea Good Clinical Practice
KOQOL: Korean version of Obesity-related Quality of Life
LOCF: Last-observation-carried-forward (analysis)
MFDS: Ministry of Food and Drug Safety
PP: Per-protocol
QSCCII: Questionnaire for Sasang Constitution Classification II
SD: Standard deviation
SOP: Standard Operating Procedure
SRI: Stress Reaction Inventory
SRRS: Social Readjustment Rating Scale
WHR: Waist-to-hip ratio
