# Supplementary material for: Euiiyin-tang in the treatment of obesity: study protocol for a randomised controlled trial
Source: Trials. 2017 Jun 21;18:289. doi: 10.1186/s13063-017-2039-8 (PMC5480117; doi:10.1186/s13063-017-2039-8)
Supplement: Supplementary file 2 — WHO Data Set. Trial information according to WHO Trial Registration Data Set. (PDF 104 kb) [file 13063_2017_2039_MOESM2_ESM.pdf]

Data Category	Information
Primary Registry and Trial Identifying Number	ClinicalTrials.gov<br>NCT01724099
Date of Registration in Primary Registry	November 2, 2012
Secondary Identifying Numbers	CCRG_2012_HP001
Source(s) of Monetary or Material Support	Korea Health Industry Development Institute
Primary Sponsor	Korea Health Industry Development Institute
Secondary Sponsor(s)	NA
Contact for Public Queries	Chunhoo Cheon, KMD<br>82-2-961-9278<br>Hreedom35@gmail.com
Contact for Scientific Queries	Seong-Gyu Ko, MD, Ph.D, MPH<br>82-2-961-0329<br>epiko@khu.ac.kr
Public Title	Evaluating Safety and Efficacy of Euiiyin-tang on Obesity
Scientific Title	Evaluating Safety and Efficacy of Euiiyin-tang on Obesity: study protocol for a randomized controlled trial
Countries of Recruitment	Republic of Korea
Health Condition(s) or Problem(s) Studied	Obesity
Intervention(s)	Active comparator: Euiiyin-tang<br>Placebo comparator: Placebo
Key Inclusion and Exclusion Criteria	Inclusion Criteria:<br>Women aged 18-65 years old<br>Patients applying to one of the followings 2.1. BMI 30kg/m2 or over 2.2. BMI 27-29.9kg/m2 with hypertension in a proper treatment and blood pressure controlled (SBP ≤ 145mmHg, DBP ≤ 95mmHg) 2.3. BMI 27-29.9kg/m2 with non-insulin-dependent diabetes mellitus and fasting blood glucose < 7.8mmol/L(140mg/dL) 2.4. BMI 27-29.9kg/m2 with hyperlipidemia in a proper treatment 2.5. BMI 27-39.9kg/m2 and Total cholesterol 236mg/dL or over or Triglyceride 150mg/dL or over at screening<br>Agreed to low-calorie diet during the trial<br>Written informed consent of the trial<br><br>Exclusion Criteria:<br>Endocrine disease such as hypothyroidism, Cushing's syndrome, etc.

	Heart disease (heart failure, angina pectoris, myocardial infarction)<br>Uncontrolled hypertension (SBP > 145 mmHg or DBP > 95 mmHg)<br>Malignant tumour or lung disease<br>Cholelithiasis<br>Severe renal disability (SCr > 2.0 mg/dL)<br>Severe liver disability (2.5 fold of normal high range value on Alanine Aminotransferase [ALT], Aspartate Aminotransferase [AST], alkaline phosphatase)<br>Non-insulin-dependent diabetes mellitus and fasting blood sugar 7.8mmol/L (140 mg/dL) or over<br>Narrow angle glaucoma<br>History or existence of neurological or psychological disease (schizophrenia, epilepsy, alcoholism, drug addiction, anorexia, bulimia, etc.)<br>History of stroke or temporary ischemic cardioplegia<br>History or existence of eating disorder such as anorexia nervosa or bulimia nervosa, etc.<br>Use of medication that could have effect on weight within last 3 months (appetite suppressant, laxative, oral steroid, thyroid hormone, amphetamine, cyproheptadine, phenothiazine or medication having effect on absorption, metabolism, excretion)<br>Use of β--blocker or diuretic as hypertension medication within last 3 months<br>Use of medication for central nervous system or central active weight reduction medication<br>Forbidden treatment (Insulin, hypoglycemic agent, antidepressant, antiserotonin agent, barbiturate, antipsychotic, medication concerns of abuse)<br>Difficult to measure anthropometric dimensions because of anatomical change such as resection<br>Surgical history for weight reduction; bariatric surgery, etc.<br>Unable to follow instructions of the trial as judged by investigator<br>Women who were pregnant, lactating, planning a pregnancy or women of childbearing age who do not agree to proper contraception (birth-control pill, hormone implant, IUD, spermicide, condom, abstinence, etc.) (Women of childbearing

	age indicate within 2 years of menopause who did not receive hysterectomy, bilateral tubal ligation, bilateral oophorectomy, etc.)<br>Use of other investigational product within last 1 month<br>Reduction over 10% of the previous weight within 6 months<br>Decided to stop smoking within last 3 months; however, keeping irregular smoking habit
Study Type	Interventional<br>Allocation: randomized<br>Intervention model: parallel assignment<br>Masking: double blind (subject, investigator)
Date of First Enrollment	December 2012
Target Sample Size	160
Recruitment Status	Recruiting
Primary Outcome(s)	Weight reduction
Key Secondary Outcomes	C-reactive protein, Blood pressure, ,Blood glucose,<br>Waist/hip ratio, Waist circumference,<br>Korean Obesity-related Quality of Life scale,<br>Korean version of Eating Attitudes Test-26<br>Total cholesterol, Triglyceride, Visceral fat area
