# Supplementary material for: Euiiyin-tang in the treatment of obesity: study protocol for a randomised controlled trial
Source: Trials. 2017 Jun 21;18:289. doi: 10.1186/s13063-017-2039-8 (PMC5480117; doi:10.1186/s13063-017-2039-8)
Supplement: Supplementary file 3 — Informed Consent Form. Example of Informed Consent Form for Euiiyin-tang trial. (PDF 69 kb) [file 13063_2017_2039_MOESM3_ESM.pdf]

[Apendix] Informed Consent Form (ver 1.5)

Study Title: Evaluating efficacy and safety of Euiiyin-tang in obesity patients: a randomized, double-blinded, placebo-controlled trial

I have read the foregoing information, or it has been read to me. I have had the opportunity to ask questions about it and any questions that I have asked have been answered to my satisfaction. I was also informed that I can withdraw the agreement and receive appropriate treatment if any adverse event occurs.
I agree that I will cooperate with study investigators and talk to study investigators about any side effects that I have while taking part in the study.
I consent voluntarily to participate as a participant in this research.
A copy of this ICF has been provided to the participant.

Participant

Print Name ___________________ Signature ____________________ Date _____________________

Legal representative (if necessary)

Print Name ___________________ Signature ____________________ Date _____________________

(Relationship)

Witness (if necessary)

Print Name ___________________ Signature ____________________ Date _____________________

Researcher/person taking the consent

Print Name ___________________ Signature ____________________ Date _____________________
